# Histaminergic afferent system in the cerebellum: structure and function

**DOI:** 10.1186/2053-8871-1-5

**Published:** 2014-06-16

**Authors:** Bin Li, Jing-Ning Zhu, Jian-Jun Wang

**Affiliations:** Department of Biological Science and Technology and State Key Laboratory of Pharmaceutical Biotechnology, School of Life Sciences, Nanjing University, Mailbox 426, 22 Hankou Road, Nanjing, 210093 China

**Keywords:** Histamine, Cerebellum, Hypothalamocerebellar projections, Vestibular nucleus, Motor control, Ataxia

## Abstract

Histaminergic afferent system of the cerebellum, having been considered as an essential component of the direct hypothalamocerebellar circuits, originates from the tuberomammillary nucleus in the hypothalamus. Unlike the mossy fibers and climbing fibers, the histaminergic afferent fibers, a third type of cerebellar afferents, extend fine varicose fibers throughout the cerebellar cortex and nuclei. Histamine receptors, belonging to the family of G protein-coupled receptors, are widely present in the cerebellum. Through these histamine receptors, histamine directly excites Purkinje cells and granule cells in the cerebellar cortex, as well as the cerebellar nuclear neurons. Therefore, the histaminergic afferents parallelly modulate these dominant components in the cerebellar circuitry and consequently influence the final output of the cerebellum. In this way, the histaminergic afferent system actively participates in the cerebellum-mediated motor balance and coordination and nonsomatic functions. Accordingly, histaminergic reagents may become potential drugs for clinical treatment of cerebellar ataxia and other cerebellar disease. On the other hand, considering the hypothalamus is a high regulatory center for autonomic and visceral activities, the hypothalamocerebellar histaminergic fibers/projections, bridging the nonsomatic center to somatic structure, may play a critical role in the somatic-nonsomatic integration.

## Introduction

The cerebellum is a well-known important subcortical motor structure, ensuring coordination, precision, and accurate timing of movement, and learning motor skills [1–4]. The cerebellar neuronal circuitry, organized elaborately and modularly, receives two major types of afferent inputs, mossy fibers and climbing fibers [4, 5]. The former originates from nuclei in the spinal cord and brainstem and carries sensory information from the periphery as well as information from the cerebral cortex, while the latter originates from the inferior olivary nucleus and sends error signals sensed from the motor performance of periphery musculatures to the cerebellum. In addition to obtaining specific and discrete information from the mossy and climbing fiber afferent systems, the cerebellum also receives nonspecific signals from the so-called third type of afferents, typically beaded fibers [6], which contain various amines or neuropeptides. Although more than 20 different types of amines and neuropeptides, such as serotonin [7], norepinephrine [8], histamine [9], orexin [10], and CRF [11, 12], have been found in the cerebellum, their functional significance is largely unknown. In general, beaded fibers form varicose contact with Purkinje cells and interneurons in the cerebellar cortex, as well as neurons in the cerebellar nuclei, fastigial (FN), interpositus (IN) and dentate (DN) nuclei, and exert a widespread modulatory role in the cerebellar circuitry [2, 6, 13].

Monoamines are firstly identified neurotransmitters used in the third type of afferents in the cerebellum. Among them, histamine is a newly found one in the cerebellar afferents. Although histamine was isolated from peripheral tissues as a biologically active amine more than a century ago, histamine acting as a neurotransmitter in the brain and the central histaminergic system gained general acceptance only in recent 30 years [14]. Peripheral histamine is well known to be stored primarily in the tissue mast cells and enterochromaffin-like cells, and holds a pivotal position in allergic reaction, gastric acid secretion and contraction of smooth muscle tissues of the lungs, whereas central histamine tends to be considered as a “modulator for whole brain activity” [14–17]. In the cerebellum, different from serotoninergic and norepinephrinergic afferents arising from the brainstem [6, 13], histaminergic fibers originate from the hypothalamus, a higher center for nonsomatic visceral and autonomic regulation [15, 16]. In 1984, the direct hypothalamocerebellar projections were first definitively presented by Dietrichs [18] in his pioneering study on cats. A subsequent series of neuroanatomical investigations from Haines, Dietrichs, and other colleagues [19, 20] on various mammals and nonmammalian vertebrates further substantiated the direct bidirectional connections between the cerebellum and the hypothalamus, the cerebellar-hypothalamic circuits. Since the cerebellar-hypothalamic circuits extensively exist and appear to be stronger in species ascending the phylogenetic scale, the connections may be phylogenetically old pathways [19]. The neurotransmitters in the hypothalamocerebellar projections have not been well known so far, however, a growing body of data has provided strong evidence that histamine is a potential candidate and plays an important functional role in modulating activity of the cerebellar circuitry. In this review, the structure and function of hypothalamic histaminergic projections in the cerebellum are summarized and discussed.

## Review

### Origination of histaminergic afferents in the cerebellum

**Figure 1:**
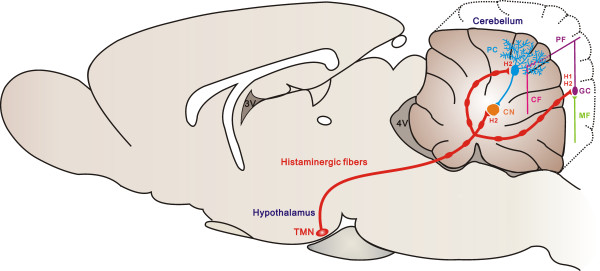
**Hypothalamic histaminergic afferents in the cerebellum.** Cerebellar histaminergic afferent fibers originate from the tuberomammillary nucleus in the hypothalamus and project to both of the cerebellar cortex and nuclei. They parallelly modulate the Purkinje cells, granule cells and nuclear neurons via H2 and/or H1 receptors and sequentially influence the outputs of the cerebellum. CF, climbing fiber; CN, cerebellar nuclei; GC, granule cell; H1, histamine H1 receptor; H2, histamine H2 receptor; MF, mossy fiber; PC, Purkinje cell; PF, parallel fiber; TMN, tuberomammillary nucleus.

In the cerebellar-hypothalamic circuits, the direct hypothalamocerebellar projections arise from widespread nuclei/regions in the hypothalamus, including the lateral, posterior, and dorsal hypothalamic areas, the dorsomedial and ventromedial nuclei, the periventricular zone/nucleus, the lateral mammillary and supramammillary nuclei, as well as the tuberomammillary (TMN) nucleus [17, 19]. Using an immunofluorescence technique, Ericson et al. [21] demonstrated Fast Blue-labeled l-histidine containing neurons in the TMN after cerebellar injections. In fact, series of studies have ascertained that the TMN is not only the origination of hypothalamocerebellar histaminergic afferents (Figure 1), but also the specific sole region of origin for the whole central histaminergic system in the brain [14, 16].

The TMN is a small nucleus located in the posterior hypothalamus. The histaminergic neurons in the TMN mostly have large somata (20–30 μm diameters) with resting potential of about -50 mV. These neurons are spontaneously active with slow regular firing rate at 1–4 Hz and mean mid-amplitude duration of action potential at 1–3 ms [22]. Although hyperpolarization-activated cyclic nucleotide-gated (HCN) channels are expressed in histaminergic neurons, they are not responsible for maintaining the neuronal spontaneous activity as a pacemaker. A complex mechanism involving Na^+^, K^+^ and Ca^2+^ conductances contributes to the pacemaker properties [14–16]. Importantly, the firing rate and pattern of histaminergic neurons varies in different behavioral states, with a ratio of 1.5 between firing rates of histaminergic neurons in active and quiet waking in the cat [23, 24], suggesting the central histaminergic system is closely related to not only wakefulness but also movement.

### Innervation of histaminergic afferents in the cerebellum

By means of immunocytochemistry using anti-histidine decarboxylase (HDC, the enzyme catalyzing the reaction that produces histamine) antibody or antiserum against histamine, the detailed distribution of histaminergic fibers in the cerebellum has been successively examined in the guinea pig, rat, tree shrew, and human [9, 25–27]. In the rat cerebellum, HDC-immunoreactive fibers are scattered in all three cerebellar cortical layers, the molecular, Purkinje, and granular layers, rather than concentrated in any specific region [25]. However, other studies did not find any histaminergic afferents in rat cerebellum [28] or very low density in the cerebellar cortex [29]. Similar to those in the rat, the histaminergic fibers are sparsely distributed in all cortical layers in the guinea pig cerebellum, with more denser fiber networks in the vermis and flocculus and less fiber density in the cerebellar nuclei [26]. However, more histamine-immunoreactive fibers innervate cerebellar nuclei in the tree shrew [27]. In human cerebellar samples, a moderate density of histaminergic afferents has also been observed in the molecular layer, and more fibers have been seen in the granular cell layer. Additionally, these fibers run parallelly to the Purkinje cell layer after traversing it perpendicularly [9].

The histaminergic fibers share many morphological similarities, including distribution, orientation, branching patterns, and ending sites, with the serotoninergic, noradrenergic and neuropeptidergic axons in the cerebellar cortex (Figure 1). On the basis of these structural properties, the histaminergic afferent fibers in the cerebellar cortex are considered to be classified as multilayered fibers. Furthermore, the most endings of histaminergic fibers do not make typical synaptic specializations but form varicosities. The varicose rather than synaptic contact pattern, together with the dispersive innervation of hypothalamic histaminergic afferents in the cerebellar cortex and nuclei, indicates an extensively modulatory role of histamine in the cerebellar circuitry.

In the TMN neurons, histamine is synthesized from l-histidine through oxidative decarboxylation by HDC. Then, histamine is stored in neuronal somata and especially in axon varicosities, where it is carried into vesicles through the vesicular monoamine transporter VMAT-2 and released in a calcium-dependent manner upon arrival of action potentials [14]. In the targets, histamine is inactivated through transfer of the methyl group from *S*-adenosylmethionine by histamine *N*-methyltransferase (HMT) or via oxidative deamination by diamine oxidase (DAO). However, HMT rather than DAO terminates histaminergic transmission in the cerebellum, since only HMT is expressed in the cerebellum [30]. Inhibition of histamine methyltransferase enhance phosphoinositide turnover in the cerebellum [30], which is mediated by histamine H1 receptors.

### Expression and distribution of histamine receptors in the cerebellum

Up to date, four histamine receptors, H1-H4 receptors, have been cloned and identified, in which H1, H2 and H3 receptors are richly expressed in the central nervous system [14–16]. Although histamine H4 receptors are detected predominantly in the periphery, recent studies have also reported a functional expression of H4 receptors in human and rodent brain [31–33]. All histamine receptors are metabotropic and belong to the rhodopsin-like family of G protein-coupled receptors [14–16]. Among them, histamine H1, H2 and H4 receptors are postsynaptic and mediate mostly excitatory responses, whereas H3 receptors mediate presynaptic inhibitory actions as auto- or hetero-receptors [14–16]. Owing to autoradiographic mapping, in situ hybridization and immunohistochemistry techniques, expression and distribution of histamine receptors in the cerebellum have been revealed. Accumulating evidence demonstrates that all histamine H1, H2, H3 and H4 receptors exist in the cerebellum with various species difference.

#### H1 receptor

In situ hybridization studies have revealed that histamine H1 receptor mRNAs are expressed in granular layer and Purkinje cells of the guinea pig cerebellum [34, 35]. Using [^3^H]mepyramine or [^125^I]iodobolpyramine as sensitive probe, autoradiographic mapping results have showed a high density of H1 receptors in the molecular layer of the guinea pig cerebellum [36, 37]. Substantial levels of H1 receptors have also been observed in the cerebellum of cats and mice [38, 39]. However, compared with the guinea pig, mouse and cat cerebellum, much lower level of H1 receptors are expressed in rat cerebellum [40].

#### H2 receptor

Using [^125^I]iodoaminopotentidine for radioligand binding and a ^33^P-labelled complementary RNA probe for in situ hybridization, an autoradiographic study have demonstrated that histamine H2 receptor and its mRNAs distribute in the guinea pig cerebellum, especially in Purkinje cell and granular layers [41]. Nevertheless, in the rat brain, only low level of H2 receptor mRNA expression has been detected in the cerebellum by northern blot hybridization [42]. Interestingly, in the mouse cerebellum, from developmental point of view, H2 receptor mRNA levels present an increased tendency with age [43]. The expression and location of H2 receptors have also been observed in the dentate nucleus of human and monkey cerebellum [44].

#### H3 receptor

Histamine H3 receptor, located on the somata and axon terminals of histaminergic neurons, was identified as a presynaptic autoreceptor in the rat brain by Arrang et al. in 1983 [45]. Besides acting as a presynaptic autoreceptor to modulate histamine synthesis and release, H3 receptor can also exert as a presynaptic heteroreceptor to inhibit the release of various other neurotransmitters [46], such as noradrenaline, acetylcholine, glutamate and GABA. The expression and distribution of H3 receptors in the cerebellum were observed in rodents, pigs and humans [47–49]. In rats, using a ^33^P-labelled riboprobe for in situ hybridization, a strong mRNA expression of H3 receptor, probably the shorter isoform [50], was found in most Purkinje cells as well as in the cerebellar nuclei, including the FNs and INs [48]. But there was scarce or very low detectable binding of H3 receptors in the Purkinje cells indicated by r-[^3^H]α-methylhistamine or [^125^I]iodoproxyfan for autoradiography [48, 49], suggesting H3 receptors are expressed on efferent projections rather than somata or dendrites of the Purkinje cells in rats. Furthermore, immunohistochemical analysis using affinity-enhanced anti-H3 (349–358) antibodies demonstrated that high levels of H3 receptors were detected in Purkinje cell layer but low levels in granule layer of the mouse cerebellum [47], whereas high mRNA expression of the receptors was observed in the guinea pig [51]. By PET, low binding of H3 receptors with [^11^C]GSK189254 radioligand was also detected in human and pig cerebellum [52, 53]. These observations indicate that H3 receptor expression in the cerebellum varies among species.

#### H4 receptor

H4 receptor, the newly identified histamine receptor, is expressed predominantly in peripheral tissues and cells, such as blood, lung, gut and liver [14, 54]. However, the expression and localization of H4 receptor in the brain remain controversial in different reports [32, 55, 56]. By using RT-PCR technique, Nakamura et al. reported that expression of H4 receptor mRNAs was not detected in the brain [55]. While, RT-PCR results from other laboratories demonstrated an expression and distribution of H4 receptor mRNAs in various brain regions, including high level expression in rat cerebellum [32] and mouse cerebellar granule layer [31], and low level in human cerebellum [56]. The exact expression and distribution of H4 receptors in the cerebellum still needs to be further studied.

### Histaminergic modulation on cerebellar neuronal activities

Innervation of hypothalamic histaminergic afferents on cerebellar cortex and nuclei and expression of histamine receptors in cerebellar neurons strongly suggest that histaminergic afferents may hold a key functional position in the cerebellar neuronal circuitry. In fact, a growing body of data has provided substantial evidence that histamine excites cerebellar neurons [57–63]. Although the distribution of histaminergic afferents in the rat cerebellar cortex seem to be scattered or low, electrophysiological studies show substantial evidence that histamine increases neuronal activities in cerebellar cortical circuit in rats. In 1999, Li et al. first reported that histamine induced an excitation on rat cerebellar granule cells [57], the interneurons relaying mossy fiber inputs via parallel fibers to Purkinje cells. In addition, histamine was found to excite Purkinje cells [63], the principle neurons in cerebellar cortical circuit, as well as neurons in the cerebellar nuclei, including the FN [58, 60], IN [59] and DN [62]. Interestingly, the effects of histamine on these cerebellar neurons are uniform postsynaptic excitation with various underlying receptor mechanisms (Figure 1). H2 receptors mediate the histamine-induced excitation on Purkinje cells and cerebellar nuclear neurons in rats [58–60, 62, 63], whereas H1 and H2 receptors co-mediate the excitatory effect of histamine on granule cells with a predominant contribution of H1 receptors [57]. Activation of H1 receptors in guinea pig cerebellum was also found to increase intracellular Ca^2+^ concentration in Purkinje cells [64]. Although H3 and H4 receptors are expressed in the cerebellum, role of them in histaminergic modulation on cerebellar neurons remains largely unclear up to date. It is only reported that H3 receptors inhibit and H2 receptors facilitate noradrenaline release in the cerebellum in guinea pigs [65].

It has been well known that histamine H1 receptor is coupled to G_q/11_ protein and phospholipase C (PLC), whereas G_s_ and protein kinase A underlies H2 receptor [14–16]. Following H1 receptor activation in neurons in other brain areas, leak potassium channels are blocked, or Ca^2+^-activated cation channels and/or Na^+^-Ca^2+^ exchangers are activated [14–16]. On the other hand, activation of H2 receptors in dorsal lateral geniculate relay neurons and hippocampal pyramidal cells enhances the hyperpolarization-activated cation current (I_h_) and/or inhibits a calcium-activated potassium conductance [14–16]. The whole downstream signal transduction pathways of histamine receptors in different cerebellar cortical and nuclear neurons and the underlying ionic mechanisms have not yet been revealed.

On the other hand, histamine may influence cerebellar neuronal activity through its actions on the cerebellar glial cells. It is reported that H1, H2 and H3 receptors are all expressed in the cerebellar astrocytes [66, 67], including Bergmann glial cells [68]. And histamine elevates several biochemical properties of astrocytes in the cerebellum, such as the activities of ornithine decarboxylase and glutamine synthetase, and incorporation of [^3^H]thymidine into DNA, and thus regulates growth and development of astrocytes [69]. Moreover, by using fura-2-based Ca^2+^ imaging, histamine was found to induce calcium entry in rat cerebellar astrocytes [70].

Intriguingly, besides cerebellar neurons, histamine also excites cerebellar target structures, in which vestibular nuclear complex in the brainstem plays a critical role in control of muscle tone and posture [71, 72]. The vestibular nuclear complex comprises four main nuclei, lateral (LVN), medial (MVN), inferior (IVN), and superior (SVN) vestibular nucleus. All of these four nuclei receive direct hypothalamic histaminergic innervations [73–75] and express histamine receptors [41, 48, 76, 77]. In consistent with the effect of histamine on cerebellar neurons, histamine induces an excitatory response of the neurons in vestibular nuclei. Extracellular recordings and whole-cell patch-clamp recordings in vitro showed that histamine directly excited MVN, SVN, and IVN neurons via postsynaptic H1 and H2 receptors [78–80] and depolarized LVN neurons through H2 receptors [81]. Na^+^-Ca^2+^ exchangers coupled to H1 receptors and HCN channels linked to H2 receptors contribute to the histamine-induced depolarization on MVN neurons [81, 82]. Presynaptic H3 receptor also holds a key position in vestibular nuclear circuit [83, 84] and even in vestibular compensation [83–85], however, its role in modulation of vestibular nuclear neuronal activity has not been reported.

It is noteworthy that the actions of histamine on cerebellar cortical and nuclear neurons as well as vestibular nuclear neurons are homogeneous excitation. Thus, the hypothalamic histaminergic afferent system acts to uniformly and parallelly excite components in the cerebellar circuitry as well as the cerebellar target structure, vestibular nuclear complex. Due to histaminergic varicose endings and histamine metabotropic receptors, the hypothalamic histaminergic afferent system may not transmit fast signals, but act as a biasing force to influence electrophysiological properties of cerebellar and vestibular neurons and hold their excitability and sensitivity at an appropriate level for responding to inputs coding changes in internal and external environments. In this way, the histaminergic afferent inputs may extensively modulate the sensorimotor integration in the cerebellar and vestibular circuits and sequentially influence cerebellar-related motor behaviors.

### Physiological function of histaminergic afferents in the cerebellar-related behaviors

**Figure 2:**
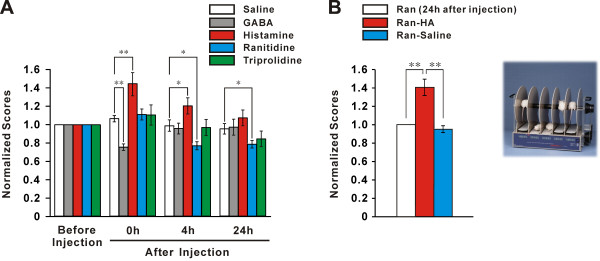
**Histamine promotes motor balance and motor coordination in accelerating rota-rod via H2 receptors in the cerebellar interpositus nuclei. (A)** Motor performances of rats microinjected with normal saline, GABA, histamine, ranitidine (antagonist for H2 receptor) and triprolidine (antagonist for H1 receptor) in accelerating rota-rod. **(B)** Reversal effect of histamine on ranitidine-injected rats. *P < 0.05; **P < 0.01. Modified from Song et al., Neuroscience, 140:33–43, 2006.

**Figure 3:**
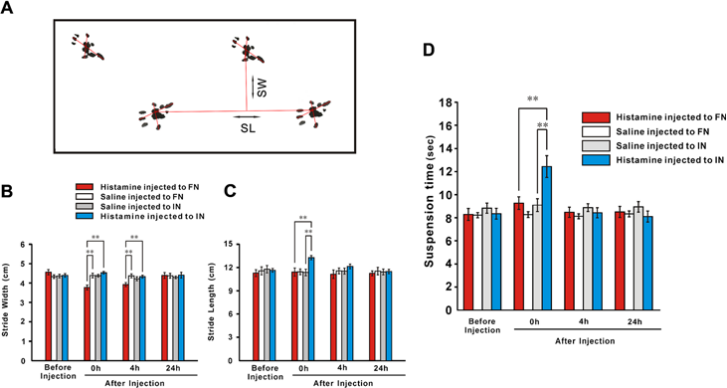
**Histamine precisely modulates trunk, proximal and distal muscles through the cerebellar fastigial and interpositus nuclei, respectively. (A)** Walking track of hindfeet of a normal rat. **(B)** Microinjection of histamine into the fastigial rather than interpositus nuclei induced a narrower stride width. **(C)** Microinjection of histamine into the interpositus but not fastigial nuclei induced a longer stride length. **(D)** Microinjection of histamine into the interpositus but not fastigial nuclei increased the endurance time of suspension. **P < 0.01. SL, stride length; SW, stride width. Modified from He et al., Behav Brain Res., 228:44–52, 2012.

The central histaminergic nervous system has been implicated in many nonsomatic basic physiological functions, such as sleep-waking cycle, energy and endocrine homeostasis, synaptic plasticity, and learning [14–16]. Recently, role of histamine and histaminergic system in somatic motor control receives increasing attention. Intraventricular administration of histamine produced a biphasic effect in spontaneous locomotor activity with an initial transient hypoactivity followed by hyperactivity [86, 87]. Depletion of brain histamine or knockout of histamine receptors influenced motor behaviors [88–90]. The activity levels, such as wheel-running and spontaneous locomotion, in the HDC knock-out mice were lower than those in the wild types [91]. Knockout of H1 receptors in mice altered ambulatory activity and reduced exploratory behavior [89]. The H3 receptor-deficient mice showed a decrease in overall locomotion, wheel-running behavior, and stereotypic responses [90]. Interestingly, bilateral microinjection of histamine into the cerebellar FNs or INs, two final output nuclei of the spinocerebellum, does not influence overground locomotion in rats in an open field [58, 61]. However, microinjection of histamine into the FNs and INs significantly lengthens the endurance time of rats on an accelerating rota-rod (Figure 2) and shortens the time that rats spend traversing a balance beam, which is mediated by H2 receptors [58, 61], indicating a promotion of histamine on cerebellum-mediated motor balance and motor coordination. Furthermore, microinjection of histamine into bilateral FNs narrowed stride width of footprint but did not influence wire suspension, whereas microinjection of histamine into bilateral INs increased stride length and promoted suspension [58] (Figure 3), suggesting that cerebellar histaminergic afferent system may precisely modulate trunk, proximal and distal muscles via biasing the FN and IN.

Besides somatic motor control, cerebellum also actively participates in many basic nonsomatic regulations and even high cognitive functions [17, 92]. Interestingly, recently, histamine has been found to be involved in the cerebellar-mediated emotional memory consolidation. Microinjection of histamine into the cerebellar vermis impairs emotional memory consolidation in mice in the elevated plus-maze [93]. The impairment is mediated by H1 rather than H2 receptors [94]. However, via H2 receptors in the cerebellum, histamine enhances memory consolidation of inhibitory avoidance learning in mice [95]. These results indicate that cerebellar histaminergic afferent system may be extensively involved in cerebellar physiological functions.

### Histamine and cerebellar ataxia

Cerebellar ataxia, a form of ataxia associated with lesions to the cerebellum, is a complex motor disturbance that involves the planning and execution of movements and reduces movement accuracy and coordination [96]. Cerebellar ataxia presents with symptoms of an inability to coordinate balance, gait, extremity, and eye movements [97]. Since histaminergic afferent system plays an important role in cerebellar functions, histaminergic reagents may become potential drugs for treatment of cerebellar ataxia. Betacerc (betahistidine, an antagonist for H3 receptor and a weak agonist for H1 receptor) ameliorates symptoms of static ataxy in patients with cerebellar ataxia [98]. Ciproxifan, a potent H3 receptor antagonist, enhances MK-801 (dizocilpine, a non-competitive antagonist for NMDA receptor) produced ataxia and motor impairment [99]. Cetirizine, selective H1 receptor antagonist, decreases the falling off latency from the rota-rod and potentiates the effects of ethanol-induced ataxia [100]. The reasons why betacerc and ciproxifan exert opposite effects on ataxias still needs further investigation, Betacerc is also a weak agonist for H1 receptors and different causes of ataxias may be account for it. Although these clinical and experimental results are very preliminary, they provide a new insight and indicate a possibility of using histaminergic reagents to ameliorate symptoms of cerebellar ataxia.

## Conclusion

Histaminergic afferent system in the cerebellum, despite being a third type of cerebellar afferents, plays an important modulatory role in the cerebellar circuitry and actively participates in the cerebellar somatic motor and nonsomatic functions. Different from the serotoninergic and noradrenergic fibers originating from lower brainstem, histaminergic afferents in the cerebellum arise from the hypothalamus, a higher center for visceral and autonomic regulation. Thus, the hypothalamocerebellar histaminergic projections bridge nonsomatic center, the hypothalamus, to somatic structure, the cerebellum. These connections and especially the histaminergic modulations may not only endow the cerebellar circuitry with an appropriate functional state, but also form a vital part of the somatic-nonsomatic integration, which is critical for generating an integrated and coordinated behavioral response to changes in internal and external environment.

Although clinical use of histaminergic reagents in the therapy for cerebellar ataxia is still in exploration, intensive studies on function and receptor and ionic mechanisms of the histaminergic modulation on cerebellar circuitry may provide a new target for clinical treatment of cerebellar ataxia.
